# Why do GPs rarely do video consultations? qualitative study in UK general practice

**DOI:** 10.3399/BJGP.2021.0658

**Published:** 2022-03-08

**Authors:** Trisha Greenhalgh, Emma Ladds, Gemma Hughes, Lucy Moore, Joseph Wherton, Sara E Shaw, Chrysanthi Papoutsi, Sietse Wieringa, Rebecca Rosen, Alexander Rushforth, Sarah Rybczynska-Bunt

**Affiliations:** Nuffield Department of Primary Care Health Sciences, University of Oxford, Oxford, UK.; Nuffield Department of Primary Care Health Sciences, University of Oxford, Oxford, UK.; Nuffield Department of Primary Care Health Sciences, University of Oxford, Oxford, UK.; Nuffield Department of Primary Care Health Sciences, University of Oxford, Oxford, UK.; Nuffield Department of Primary Care Health Sciences, University of Oxford, Oxford, UK.; Nuffield Department of Primary Care Health Sciences, University of Oxford, Oxford, UK.; Nuffield Department of Primary Care Health Sciences, University of Oxford, Oxford, UK.; University of Oslo, Oslo, Norway.; Nuffield Trust, London, UK.; Leiden University, Leiden, the Netherlands.; University of Plymouth, Plymouth, UK.

**Keywords:** PERCS framework, primary care, qualitative research, remote consultation, telephone consultations, video consultations

## Abstract

**Background:**

Fewer than 1% of UK general practice consultations occur by video.

**Aim:**

To explain why video consultations are not more widely used in general practice.

**Design and setting:**

Analysis of a sub-sample of data from three mixed-method case studies of remote consultation services in various UK settings from 2019–2021.

**Method:**

The dataset included interviews and focus groups with 121 participants from primary care (33 patients, 55 GPs, 11 other clinicians, nine managers, four support staff, four national policymakers, five technology industry). Data were transcribed, coded thematically, and then analysed using the Planning and Evaluating Remote Consultation Services (PERCS) framework.

**Results:**

With few exceptions, video consultations were either never adopted or soon abandoned in general practice despite a strong policy push, short-term removal of regulatory and financial barriers, and advances in functionality, dependability, and usability of video technologies (though some products remained ‘fiddly’ and unreliable). The relative advantage of video was perceived as minimal for most of the caseload of general practice, since many presenting problems could be sorted adequately and safely by telephone and in-person assessment was considered necessary for the remainder. Some patients found video appointments convenient, appropriate, and reassuring but others found a therapeutic presence was only achieved in person. Video sometimes added value for out-of-hours and nursing home consultations and statutory functions (for example, death certification).

**Conclusion:**

Efforts to introduce video consultations in general practice should focus on situations where this modality has a clear relative advantage (for example, strong patient or clinician preference, remote localities, out-of-hours services, nursing homes).

## INTRODUCTION

There was initial optimism that the pandemic would serve as a ‘burning platform’ to propel the UK NHS towards widespread adoption of video consultations.^1^ In March 2020, NHS England moved quickly to fund new technologies for general practices;^2^ within a month, four out of five general practices had the capability to support video consulting.^3^ Scotland had established a national video consultation service in 2017; it was scaled up rapidly when the pandemic struck.^4^ In Wales and Northern Ireland, infrastructure was less developed and the digital pandemic response less well resourced, but there were local pockets of innovation including some use of video consultation.^5^

These pandemic-driven changes built on longstanding policy enthusiasm for digital primary care.^6^^–^^8^ Yet the number of general practice appointments conducted through video consultation have remained low.^9^ In England, for example, video and e-consultations combined accounted for fewer than 0.5% of general practice consultations in December 2021.^10^ This article draws on a large dataset collected mostly in-pandemic to analyse why clinicians in general practice rarely use video consultation.

## METHOD

The study identified and analysed a sub-sample of data from three mixed-method studies conducted by the team. These are summarised in Table 1 and described in detail elsewhere.^4^^,^^5^^,^^11^ These studies had different sponsors and focus, but all sought to explain successes and failures, and understand mechanisms at individual, technological, organisational, and policy level. All involved interviews and focus groups with staff and patients (which were professionally transcribed, though not always in full, and uploaded onto NVivo), collection of documents (for example, policies), and process data (for example, number of consultations by modality), analysis of artefacts (for example, software), and surveys of staff, patients, and other stakeholders. The Scottish evaluation involved pre-pandemic ethnographic visits.^4^

**Table 1.** Sub-sample of data used in this study

**Wider research study**	**Main research question**	**Sampling frame for wider study**	**Sub-sample of data on video consultations in general practice analysed for this paper**	**Key perspectives captured in sub-study dataset**
Government-funded evaluation of ‘Near Me’ video consulting service in Scotland, 2019–2020^4^	What can we learn from a national mixed-method evaluation about the knowledge, capabilities, and infrastructures needed to support the introduction and use of video consultations?	Primary and secondary care video consultation services in all 14 health boards in Scotland before and during the pandemic	27 interviews (two patients, 16 GPs, two other primary care clinicians, one clinical director, one manager, one technology supplier, four policymakers) plus focus group of seven GPs. Ethnographic field notes from site visits	Primary care staff and patients mostly living in rural and remote areas, supplied with a government-funded video consultation service
Research Council-funded case studies of in-pandemic remote general practice in England and Wales, 2020–2021 (‘Remote by Default 1’)^11^	How can technology support assessment and monitoring of patients at a distance? How can we achieve rapid spread and scale-up of remote-by-default models of primary care? How can we strengthen the NHS to support remote health care?	Video, phone, and e-consultation services in four locality-based study sites during the pandemic	39 interviews (19 GPs, eight other primary care clinicians, four GP support staff, six managers, one clinical commissioning group director, one technology supplier). Four focus groups involving four GPs, two other clinicians, three support staff, six patients, and three technology suppliers. Notes from study of technological artefacts	Primary care staff and patients from four contrasting localities (English coastal town, English university city, diverse inner-city English borough, Welsh town and surrounding region)
Charity-funded case studies of spread and scale-up of video consultations across UK (‘Health Foundation Video Consultation study’)^5^	How have the UK’s video consulting services spread and been scaled up in the context of COVID-19? What resources are needed to support and sustain them going forward? What are the consequences of rapid scale-up in times of crisis?	Primary and secondary care video consultation services (individual consultations and group clinics) in 11 study sites across England, Scotland, Wales, and Northern Ireland during the pandemic	10 primary care clinician interviews (nine GPs, one advanced nurse practitioner), 10 patient interviews, and two focus groups with 15 patients (most patient discussion of video related to their experiences in secondary care)	Primary care staff and patients selected from across UK to obtain mix of urban/rural, affluent/deprived, professional role, and experience of video consulting
TOTAL SAMPLE			55 GPs 11 other primary care clinicians Nine managers or directors Four support staff Four national policymakers Five technology industry 33 patients	

From a large combined primary dataset, a sub-sample of data was created from 121 participants on individual video consultations — including why some people did not do them (Table 1). Researchers on the three different primary studies, each of whom was familiar with the data they had collected, selected key sources (for example, a memorable interview); the NVivo database was also searched for the term ‘video’ and assessed hits for relevance. A sub-study of video group consultations will be presented in a separate paper.

**Figure 1.**
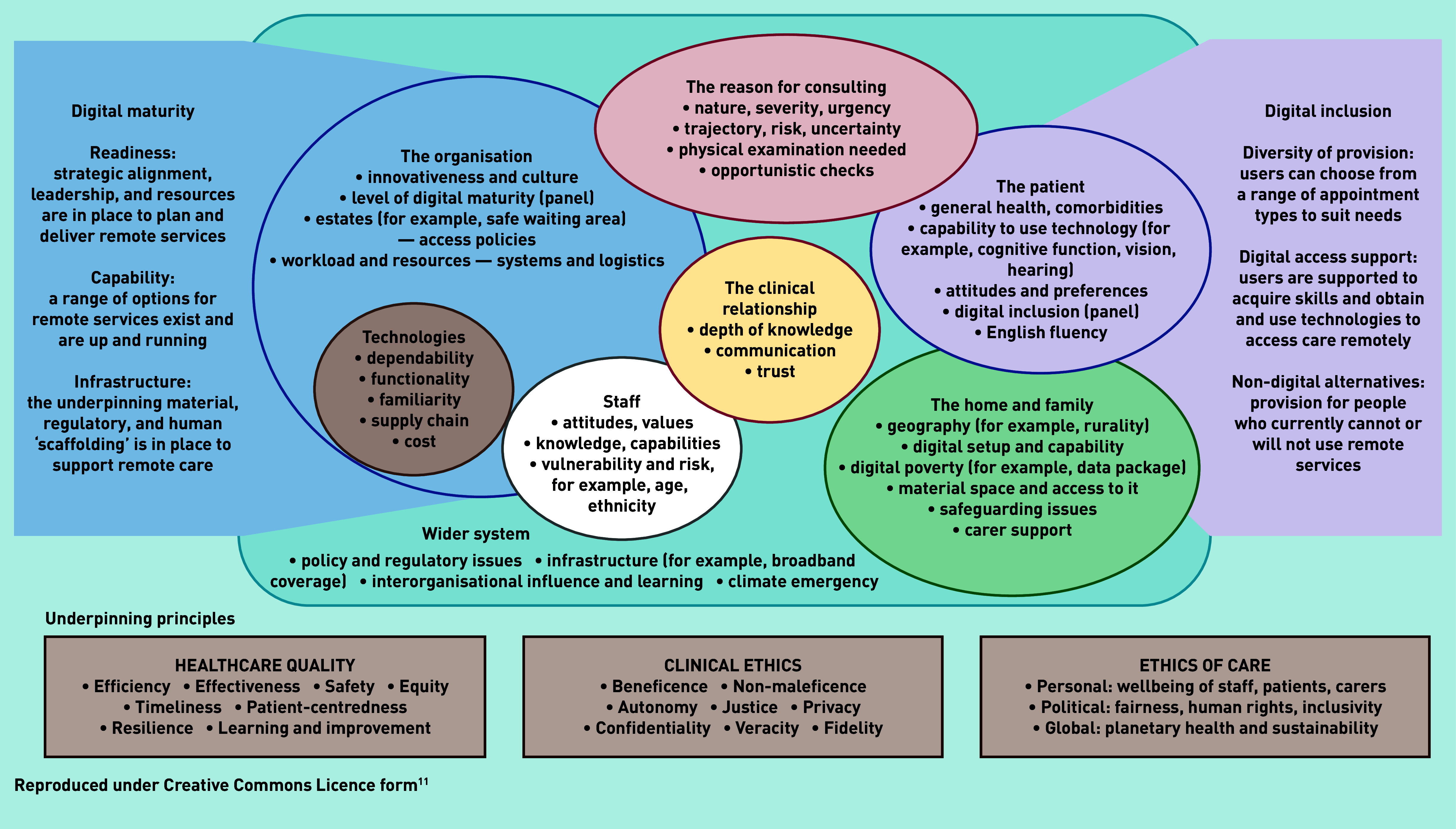
*Planning and Evaluating Remote Consultation Services (PERCS) framework.*

All sources in the sub-sample were closely read, then coded extracts using the PERCS (Planning and Evaluating Remote Consultation Services) framework (Box 1 and Figure 1).^11^ Rogers’ diffusion of innovations theory (see Discussion section) was invoked to explain the dominant theme of individual clinician resistance.^12^

**Box 1.** Planning and Evaluating Remote Consultation Services (PERCS) — explanation of domains

The PERCS framework (Figure 1), whose development and rationale is explained in detail elsewhere,^11^ is an adaptation of a more generic framework for considering the complexities involved when introducing new technologies.^13^ PERCS consists of eight interdependent domains: *The reason for consulting* covers the illness or condition and why the patient wishes to be seen (or why the clinician wishes to see them) now*.* It considers the urgency, rate of progression, whether the appointment is patient or clinician initiated, and what advice or treatment is being requested. *The patient* includes attitudes towards illness and remote consulting, which are influenced by their identity, values, personality traits, beliefs, health and digital literacy, and lived experience of illness or disability. *The clinical relationship* includes the level of mutual trust and positive regard (often though not always linked to duration of relationship) and how well the clinician and administrative team know the patient. *The home and family* includes how the material features, physical layout, symbolic spaces, and interpersonal dynamics of the home influence whether and how the patient consults remotely. People who are disadvantaged may have no home, or one that is small, crowded, lacking privacy, or not digitally connected. Family members may support — or block — the patient’s digital access. *Technologies* includes the functionality, technical performance, and ease of use of key technologies as well as their dependability and familiarity. It also covers the technology’s supply chain and its maintenance and repair. *Staff* embraces staff attitudes (grounded in professional norms and values including those relating to quality and safety of care), their digital literacy and confidence, vulnerability to infection, and levels of exhaustion. Aspects of staff members’ home environment may be relevant if working from home. *The healthcare organisation* includes innovativeness, readiness, and normalisation efforts. Innovative organisations tend to be large, well led, non-hierarchical, and with adequate slack (people and resources that can be channelled into new projects).^14^ Readiness for innovation requires both top- and middle-management support, absence of opponents, and assessment of innovation-system fit (for example, a business case). Normalisation includes supporting staff to make sense of a new technology in the context of their work; engaging them to participate; coordinating efforts to implement; and monitoring benefits and costs.^15^ *The wider system* includes the policy context (for example, technology-enabled care, planetary health, social and digital inequalities) and infrastructural elements such as broadband availability. It also includes opportunities for interorganisational influence and learning (early-adopting organisations pass on insights and resources to those coming on stream later).^14^ These domains interact and evolve dynamically over time. The PERCS framework also includes two side panels — *digital maturity* of the organisation^16^^–^^19^ and *digital inclusion* for the population it serves.^20^^–^^25^ The domains of the framework are underpinned by the principles of healthcare quality,^26^ clinical ethics,^27^ and the ethics of care more widely.^28^

**Table:** How this fits in

The pandemic provided strong impetus to extend remote consultation services in general practice, but video remains infrequently used. This study used in-depth case study methods to explore the multiple interacting influences on the non-adoption and abandonment of video consulting in general practice. Telephone was considered adequate for most remote consultations; the need for a hands-on physical examination explained why video rarely replaced in-person assessment in the remainder.

## RESULTS

As Table 1 illustrates, the dataset was drawn from primary care clinicians, patients, and other stakeholders living in all parts of the UK and representing the full range of settings and jurisdictions. It is worth noting that, despite this diversity, the similarities in findings across settings were more striking than any differences between them — notwithstanding some atypical counter-examples (illustrated in Box 2 and discussed in the text further on).

**Box 2.** Counter-examples where video added value

The examples below show that some clinicians had used and valued the video format in situations where many interviewees had said it did not add significant value.
**Out-of-hours care**
Particularly in out-of-hours contexts (where pre-threshold probability of pathology was higher and level of risk greater), some GPs used video as an adjunct to telephone for rapid visual assessment (‘eyeballing’), especially for children. Video was also used for reassurance and following up problems that had not resolved: *‘I can see on their* [parents’] *faces whether they’ve been reassured by my description of why I’m not worried about their kid’s breathing etc. and I know whether I can leave it there or whether they’re going to end up needing to come in anyway.’* (GP interview [RBDGP-CH1])
**Nursing homes**
Video was deemed helpful for linking with nursing home staff to discuss complex patients [HFVC19-GPW], ‘eyeballing’ unwell patients, especially in the pandemic context where in-person visits carried a risk of reintroducing infections to vulnerable patients, and for statutory functions: *‘The times I use it* [video] *regularly is for nursing homes — it is legally required for me to see a patient to write a death certificate and it is now allowed to see them digitally.’* (GP interview [RBDGP-NL1])
**Emergency assessment of very unwell patients**
While video did not change management in most emergencies, it sometimes provided a crucial clue: *‘I could just see how breathless she was and I was counting her respirations on the phone and, you know, I couldn’t pick it up on the phone, I spoke to her first and said, “I think let’s do a video call”, and it was like “Gosh, she’s a lot more breathless than I realised.”’* (GP interview [RBDGP-EH1])
**Talking a patient through self-examination**
While remote physical examination was often unhelpful, one GP interviewee [RBDGP-KI1] reported a trainee picking up a case of appendicitis using patient self-examination on video.
**Patients with mental health issues**
While some patients with mental health issues strongly preferred in-person contact (see main text), some were much better able to access the care they needed by video: *‘So, to be able to know that I can just sit … be at home and still have that consultation, honestly it’s amazing, and thinking back to in the past when I used to suffer from mental health difficulties, I used to cancel quite a lot of my appointments because I didn’t want to go out.’* (Interview with patient (remote locality) [HFVC02])
**Less experienced clinicians**
Some trainees and early-career GPs or trainees, who were familiar and confident with new technology, reported using video, rather than telephone, to compensate for their limited knowledge of the patient and limited experience managing risk in general practice.
**Chronic disease check-ups**
Some nurses used video to help assess lifestyle and coping in chronic disease self-management: *‘You get a bit more from patients if you can actually see them as well. One* [chronic disease review] *that I did, the patient was having a cigarette while I talked to him!’* (Nurse interview [RBDPN-MS1])
**Patients with communication challenges**
All interviewees agreed that telephone was unhelpful for the hard-of-hearing and limited English speakers. Some felt that video could be a worthwhile alternative to in-person contact for such patients: *‘A video could be quite useful with language barriers because you can, you can kind of see the gesticulations and things like that a bit, a bit more easily, so I might do a video quickly if, if I wasn’t sure about the history. And I think you’d quickly see how much discomfort he would be in.’* (GP in clinician focus group [RBDFG1-R1])

A few clinicians in the sample worked in practices where video consultations had become ‘business as usual’. But most had never tried video consultation and others had used it only briefly. Below, the multiple interacting reasons for the widespread non-adoption and abandonment of this modality in general practice were considered.

### The reason for consulting

The interviewees commented that general practice consultations cover a vast range of conditions, concerns, levels of severity, and kinds of risk. Many are unstructured, and even structured formats (for example, oral contraceptive checks) may unfold in an emergent and non-linear way as additional patient concerns or opportunistic checks are brought in. In contrast, they felt, secondary care consultations typically cover a narrow range of illnesses and are often highly structured.

A clinician-manager with a strategic role in remote consultation services reflected that, whereas surgical consultations tend to be ‘hands-on’ (surgeons assess patients by feeling) and favour in-person encounters, and physiotherapy consultations are often very visual (the therapist asks the patient to, for example, move a limb and observes them), and hence can often work well by video, general practice consultations are usually *‘conversation-oriented’* (stakeholder interview [RBDS-NH1]).

Clinicians said that, in a telephone conversation, they could often exclude a serious problem or establish a working diagnosis by taking a history and comparing it with their personal knowledge of the patient and the longitudinal medical record:

*‘I don’t need to see them to understand what they’re telling me or how they’re feeling about things, I can hear it I think.’*

(GP interview [RBDGP-KI1])

Some general practice consultations served an administrative function (for example, renewing a sick note) or a pastoral one (for example, bearing witness to suffering), both of which, many clinicians felt, could be achieved without a visual component. If further action was needed, the verbal story often provided enough information to justify a test or referral without physical examination, though some statutory functions required a visual inspection (Box 2).

Acute problems were often dealt with by non-medical clinicians (such as advanced clinical practitioners or practice nurses) who were generally comfortable providing ‘wait and see’ advice (for example, for a febrile but not unwell school-age child) on the basis of a telephone history, subject to a safety-netting plan with the option of in-person assessment.

Clinicians in general practice did not feel the need to examine every patient because, in most cases, a physical examination would not change their management. But almost every practitioner interviewed relayed a story of a patient who needed to be brought in for physical assessment when the verbal history revealed a ‘red flag’ symptom (for example, severe breathlessness) or when the conversational format afforded by the telephone proved unproductive. In such circumstances, they generally felt that a video consultation would not provide sufficient added value over an in-person encounter (*‘*[video] *is basically a phone call with the added bonus of being able to see your face.’* GP interview [RBDGP-CH2]).

Most GPs considered neither telephone nor video appropriate for acute emergencies, which (they felt) needed either an immediate ambulance or a hands-on examination: (*‘*[video] *is never useful for, you know, for when you need a proper examination so an acute abdomen or a, you know, somebody with sort of shortness of breath or chest pain.’* GP interview [RBDGP-SS1]), though counter-examples are given in Box 2.

GPs generally preferred to manage risk through in-person assessments or referring the patient on (for example, to a dedicated COVID-19 assessment hub) rather than using video.

While those with experience of video consultations had become adept at performing limited physical examinations remotely, these were often lengthy and challenging for both parties (because they involved giving and interpreting complex instructions)^29^ and clinicians disliked having to judge whether it was safe to make compromises. In most cases, an in-person examination was considered easier, quicker, and likely to produce higher-quality assessment (but see counter-examples in Box 2):

*‘At the moment our rudimentary neurological assessment on a video consultation is to get patients to walk on their toes, walk on their heels, rub their hands up and down their legs to see whether they’ve got sensation, and obviously we can’t do reflexes* [remotely]. *’*

(GP in clinician focus group [RBDFG1-R6)

### Patients’ views on video consultations

Most patients interviewed had never had a video consultation. Some were unaware that video might be an option and some knew it was not an option in their practice. Of those who had opinions, some were strongly in favour of video consultations, usually because of a positive experience with a prompt and effective appointment — though almost all examples given were of secondary care or community physiotherapy.

In patient focus groups and workshops, there was strong consensus that practices should offer a range of modalities without making assumptions about which patients or conditions would be ‘suitable’ for each approach. Some patients were opposed to video consultations because they firmly believed that in-person consultations were inherently better.

Practice staff depicted some patients as unwilling to try video consultations because they did not wish to appear on video (*‘I offer it to most people with mental health conditions and most of them say, “I’d rather just do the phone.”’* [RBD2GP EH1]) or because they viewed it as an inferior option (*‘I think people are getting more and more to wanting to go back to the old school and being able to come in, make an appointment, see their doctor.’* GP interview [HFVC19-GPW]).

Practice staff also depicted certain patient or disease groups as ‘unsuited’ to video consultation. The very old, for example, were seen as too set in their ways to adopt a new modality, though some clinicians described successful video encounters with older patients supported by family members. Video consultation was considered more risky when patient factors increased the level of uncertainty — the very young or those with low health literacy, poor technical skills, communication difficulties of various kinds, cognitive impairments, or complex multimorbidity:

*‘* [people with learning difficulties] *would need to be seen face-to-face if they need an examination. They can’t explain their pain, nor where it hurts or what it feels like or how long it’s been and also the rapport’s just not there with video consult for those people.’*

(Practice manager in support staff focus group [RBDFG2-R1])

Interviewees gave examples of patients who had been opposed to video consultation ‘come round’ to this modality after being encouraged to try it. But they also described vulnerable patients whose needs, they felt, would be met only by seeing them in person (*‘… on video he* [patient with mental health issues] *was just not coping with not real faces, he needed to see real faces.’* (Participant in support staff focus group [RBDFG2-R2]), though Box 2 gives a counter-example of a patient with mental health issues who strongly preferred video to in-person. The capacity of patients with complex needs to use video consultation could sometimes fluctuate, depending on both illness-related and social factors.

### The clinical relationship

A personal relationship characterised by positive regard, trust, and continuity over time is often celebrated as the cornerstone of high-quality general practice,^30^^,^^31^ though not all patients want or need such a relationship for every encounter. Despite a small empirical literature on ‘therapeutic presence’ in the digital environment,^32^ it remains unclear whether, in what circumstances, and to what extent the clinical relationship can be developed and maintained via video consultation. Some clinicians expressed concerns that close personal relationships with patients would not be sustained in the absence of in-person contact:

*‘We’re on a bit of borrowed time because we’ve got relationships with patients established already, and I think those will wear out over time.’*

(GP interview [HFVC04-GPW])

If in-person encounters stopped (though, in theory, video consultation might be used occasionally as part of a mixed-modality service to *maintain* an element of continuity):

*‘Is there something about emotional transference?* [In an in-person consultation] *you can clock that someone is anxious about something that’s bugging them. You can open up a dialogue. Would that be less evident in a video consultation? Unless you know someone really well so you could already pick up on those cues?’*

(GP with interest in mental health [NMGP-DD1])

Some clinicians affirmed that they did video consultations primarily for ‘patient reassurance’, and patients occasionally mentioned this as an advantage of video consultation. Some clinicians and patients felt it was unclear how their respective roles should play out in the remote consultation, resulting in stilted interactions:

*‘… maybe part of what we haven’t yet developed is a consultation etiquette for, for being online or on the phone.’*

(Participant in patient focus group [RBDFG3-R1])

Balint wrote about the patient’s ‘hidden agenda’ of socioemotional issues (including anxieties about symptoms that might indicate serious illness), which a skilled GP can help to bring to the surface.^33^ Both clinicians and patients expressed concerns that remote modalities may suppress this hidden agenda and reduce the number of what one GP interviewee called *‘doorknob diagnoses’* [RBDWS1].

### The home and family

Participants in rural areas pointed out how video consultations could save a long journey for the patient or a health professional visiting them. But they expressed concern that increasing reliance on video consultation services, many of which were accessed through smartphone apps, could create a two-tier service (which was, *de facto*, a digital-priority service) given the poverty of digital infrastructure in some locations and the limited digital set-up in some homes. The introduction of home-schooling during the pandemic had prompted more affluent families to invest in, and become familiar with, videoconferencing technology. But whereas most people can access telephone, poorer families may have limited broadband capacity and share smartphone or webcam access between several members.

### Staff attitudes

Staff attitudes towards video consultations were influenced by their own technical competence and confidence, their experience and knowledge (including personal knowledge of the patient and whether they themselves had actually tried using video consultations), their perceptions of quality and risk, the cognitive demands of different modalities, job satisfaction, and (lack of) role models.

Practice staff varied in their digital experience *,* digital literacy, and confidence. Some were described as *‘absolutely petrified’* of video consultation (nurse interview [RBD-UH1]). One participant described a video mental health support programme to which staff had more difficulty connecting than patients. While some described technology failures, most considered technical barriers minor (*‘*[video consulting] *is really quite straightforward’* (GP interview [RBDGP-EG1])).

While many clinicians mentioned medicolegal concerns (for example, fear of litigation from a missed diagnosis), most felt that choice of modality (for example, phone, video, or in-person) was driven primarily by the best interests of the patient. Clinicians varied in their tolerance of clinical risk, with some described by their colleagues as ‘anxious’ (that is, having a low threshold for defaulting to in-person consultations) and others as ‘over-confident’ (having a high threshold).

Clinicians generally found video consultations more cognitively demanding than face-to-face or telephone ones. Even when there was some visual input, the signals were more limited than an in-the-flesh encounter (one GP interviewee described it as *‘2D rather than 3D’* [RBDGP-FM1]).

Clinicians also felt that they gained less job satisfaction from video and telephone consultations than in-person ones. Older clinicians in particular talked of the sense of fulfilment going out of a job they had enjoyed all of their professional lives, because of the more limited human connection achieved remotely:

*‘We’re more stressed now than we’ve ever been. Everyone is. There’s additional stresses I know — the schools are closed and there’s the day-to-day things. But sitting there, day-to-day, doing everything by phone* […] *Just a day or two ago we started doing some face-to-face clinics again. Everyone’s much happier. They’re all commenting how much happier it is face-to-face* . *’*

Interviewer: *‘Are you using video at all?’*

*‘Yes very occasionally. It didn’t really work for us. Except we get them to send photos for dermatology. The photos are meaningful. But mostly the phone is fine.’*

(GP interview [NMGP-CY1])

Box 2 gives counter-examples of younger clinicians who were confident with video technology and felt that it helped them manage risk and interact with patients they did not know well.

One reason clinicians gave for not adopting video consultation or abandoning it was that nobody else was using this medium (*‘it’s really hard to keep being an outlier when everybody that you’re working with thinks it’s a strange way to work’* (GP who had been a video consultation enthusiast but later abandoned them [RBDGP-CH3])). ‘Champions’ for video consulting were few and far between, and some clinicians and support staff considered this modality a passing fad.

### General practices (organisation domain)

Practices varied widely in their digital maturity, the priority they gave to introducing innovations, and how much spare resource they had to implement and evaluate them. Many were overstretched and understaffed. Several participants said that their practice had invested in video technology but had not got round to installing it, training staff to use it, or developing new workflows to accommodate it into business-as-usual. In some practices, only one or two rooms were equipped for video calls so clinicians had to share them, causing bottlenecks. However, technological advances meant that, for certain products, there were few organisational hurdles.

The type of consultation offered to patients was often heavily influenced by the need to manage practice workload:

*‘We’ve tried to kind of go back to the point of, “OK what, what is the problem? What is it that you need to discuss with somebody?” and we’re not promising that they’ll even get a telephone call* […] [A] *patient phones up, reception take as much detail as possible* […] *and then the GP will read that and decide whether they want to call them, video call them, or send them a text.’*

(Practice manager interview [RBDPM-FT1])

In many practices, telephone call-backs had become the default remote option. Such calls were relatively easy for the practice to offer since patients were rarely given a specific time slot. Video consultation was seen as logistically more challenging:

*‘In secondary care, they just see the clinic list and send the patient a link* [in advance]. *Whereas for us, if we’re doing an acute review, we need to send the URL to the patient on the same day … And we see a lot of patients in a day. And the processes are complex — there are lots of areas we have to build it into: care home appointment, acute appointment, routine appointment, minor surgery, family planning, long-term condition review, and so on.’*

(GP with national quality improvement role [NMGP-TL1])

From the patient’s perspective, they found it relatively easy to step out of a meeting or find a quiet space in a shopping centre to take a phone call from their clinician, but a video call required more privacy and had to be carefully pre-arranged.

Some participants speculated about future scenarios in which video consultations might add value — for example, to enable home-working by a clinician, thereby freeing up limited practice space for essential in-person consultations. Such arrangements were not directly observed in this study.

### Technologies

Telephone is a familiar and dependable technology that almost everyone knows how to use. Video technology, on the other hand, may be unfamiliar (at least in this context); it may not work at all or it may fail to provide adequate audio or visual quality for a satisfactory encounter. Even though most video consultations were technically adequate, they were described by many GPs as ‘fiddly’ to set up and did not always provide adequate visual or audio quality (*‘I suppose because our initial consultations were so difficult, we have gone mainly telephone* . *’* GP interview, locality with poor broadband [HFVC06NI]). The fact that a substantial percentage of video consultations failed for technical reasons was considered time consuming, frustrating, and professionally embarrassing by clinicians.

Developers of bespoke video consultation software described how they had worked before and during the pandemic to make the technologies easier to use and more reliable. Key technical advances included removing the need for either party to download software, install peripherals such as webcams and headphones, or obtain and use access passwords. One company had developed a smartphone-based product that allowed the clinician to convert a telephone call to a video call without hanging up, thereby reducing double-handling. The same product allowed text, photograph, and document transfer, which significantly increased its appeal. Photographs sent by this route were considered to provide better image quality than video. These advances made video consulting operationally smoother, and policymakers depicted the novel products in plug-and-play terms, requiring little or no additional infrastructure, new routines, or training (national stakeholder interview [RBDNS-NC1]).

However, not all practices had selected these new-generation technologies (and in some settings they were constrained by a locality-wide purchase of an inferior product). Even when they had, clinicians and patients still found the telephone more reliable, quicker, and more intuitive.

### The wider healthcare system

In most of the UK, the pandemic triggered extensive healthcare innovation with rapid implementation of cross-government emergency measures to fund technology development, reduce red tape, align incentives, and support installation and use of new technologies.^5^ The very favourable policy context, along with unprecedented effort from clinicians and managers, enabled many NHS organisations to acquire the capability to deliver a video consultation service back in March 2020. But this did not translate into widespread uptake and use of video consultations, partly because, as described previously, the relative advantage of video consultation was unclear and partly because the near-absence of practices with up-and-running video services meant that there was no mimetic pressure to conform with this organisational innovation.^34^ A reviewer suggested that local rules of thumb, such as refusal of secondary care clinicians to accept referrals unless the patient has been seen face-to-face, would be a powerful disincentive to GPs using video consultation, though no examples of this were found in the dataset.

## DISCUSSION

### Summary

This qualitative analysis explored why, with few exceptions, video consulting was either never adopted or soon abandoned in UK general practice. Applying the different domains of the PERCS framework (Figure 1), this was despite many enabling wider system factors (for example, a favourable policy context, removal of many regulatory and financial barriers) and significant advances in many of the *technologies* (for example, in functionality, dependability, and usability). While residual limitations in some technologies — having what some clinicians described as ‘clunkiness’ — explained some non-adoption and abandonment, another key reason was lack of relative advantage of video consultation for dealing with the *reason for consulting* (many presenting problems could be sorted adequately and safely by telephone and an in-person assessment was considered necessary for the remainder). Video consultation sometimes added value, however, for out-of-hours and nursing home consultations and statutory functions (for example, death certification). Patients were variably capable of participating in video consultations and their views on this modality varied (some gained reassurance from seeing the GP’s face on video consultations while others found therapeutic presence was achieved only in person); patients’ home circumstances sometimes precluded the video consultation option. Staff also had variable skills, confidence, and experience in video consulting, and both staff and patients felt that the clinical relationship was more easily initiated and maintained face-to-face.

### Strengths and limitations

This study benefited from a large qualitative dataset collected at a time when, uniquely in history, the contextual preconditions for introducing video consultations were extremely positive. The PERCS framework, whose development and rationale is described in detail elsewhere,^11^ enabled multiple interacting influences on the adoption, spread, and sustainability of video consultations to be mapped. This is a rare example of a study of non-adoption and abandonment of a technological innovation, and thus helps correct a pro-innovation bias in the literature. By highlighting counter-examples (Box 2), the explanatory model was refined.

The main limitation is that pandemic restrictions made ethnography impossible (except for a brief period pre-pandemic); therefore, video consultations in general practice were not able to be directly observed. In addition, the data collection occurred in a very particular historical context (mid-pandemic, with a positive policy push). The study was not able to capture the dramatic policy reversal in the UK in September 2021 (following a media-led campaign) from ‘remote-by-default’^35^ to *‘GPs must see patients in-person if requested’*,^36^ which may have reinforced negative attitudes towards remote forms of consulting. The transferability of the findings beyond a UK setting is unknown.

### Comparison with existing literature

The findings resonate with the conclusion of previous researchers that most technological innovations introduced into healthcare settings *‘fail because, despite high investments in terms of both time and financial resources, physicians simply do not use them’*.^37^

In his book *Diffusion of Innovations*, based on hundreds of empirical studies, Rogers defined six attributes of innovations that, in the eyes of potential adopters, account for variation in the speed and extent of adoption: relative advantage, low complexity, compatibility, trialability, observability, and potential for reinvention.^12^ The most important is relative advantage: if the intended adopter sees no advantage over existing practice, they will not adopt — or will quickly abandon. In most sites in the study (Northern Ireland being an exception), technical infrastructure was adequate and some, though not all, video technologies were simple and easy to use.

GPs were able to try out these technologies and observe their impact. Video consultation was — for some consultations — compatible with professional values, standards, and ways of working. But its advantages were, for most general practice caseload, minimal, and because few clinicians were enthusiastic users there was normative pressure on clinicians to not use this innovation.

The authors have recently reviewed the extensive research literature on video consultations collected pre-pandemic;^11^ it consists mostly of experimental trials conducted in secondary care on highly selected patients with stable chronic conditions, hence has limited relevance to real-world general practice in a pandemic setting.

The findings in this study accord well with other in-pandemic studies of remote general practice services. In a detailed quantitative and qualitative study of remote care in Bristol, UK, in 2020–2021, Murphy *et al* showed that 1% of consultations overall and 3% in the over-80s occurred by video.^9^ Like the present study, they found telephone adequate for many remote consultations and in-person assessment preferred for most of the remainder. They also found that a photograph-plus-telephone was often preferred to visual examination by video consultation, but concluded that video consultation sometimes had added value for *‘children, nursing homes, multidisciplinary team meetings, and problems that require dynamic assessment’*.^9^

### Implications for research and practice

As Tudor Hart said, *‘Primary health care is doing simple things well, for large numbers of people, few of whom feel ill.’*^38^

The pandemic has shown that these ‘simple things’ can often be achieved by telephone but that in-person encounters are necessary for some patients. The video consultation option, even with modern, easy-to-use technologies, may be overly complex for the former case and a poor substitute for hands-on interaction in the latter.

As noted by a reviewer of an earlier draft, while the findings explain the current low use of video consultation in general practice, they reflect how staff and patients *currently perceive* video consultations rather than how these individuals might feel if they had a chance to try out video consultation in different kinds of consultations. This observation relates to what Rogers called the trialability of the innovation — how easy it is to try it out and see its impact without committing to adopt it.^12^ Given the current pressures on general practice, the trialability of any innovation is limited.

Rather than trying to encourage video consultations across the full caseload of general practice, this modality should be targeted, at least initially, to situations where video consultation offers a clear relative advantage. These include — but may not be limited to — situations where there is a strong preference to avoid an in-person visit (for example, remote localities, patient choice, less experienced clinician, one party shielding), when ‘eyeballing’ a patient is likely to add value to a telephone call (for example, some out-of-hours calls, nursing home virtual visits), or when a statutory visual inspection is required.

The image of remote consulting often portrayed by the media — with doctor and patient connecting via their smartphones by video link — is currently inaccurate. The old-fashioned telephone or its modern equivalent, the mobile phone, is widely used and appears fit for purpose for some but not all general practice consultations — though, somewhat ironically, there is relatively little research on this modality. The findings of this study suggest that in-person consultations remain the gold standard for many, though by no means all, clinical interactions. As general practice faces an uncertain future characterised by a continuing pandemic, rising demand, staff shortages, and cash constraints, research on how best to allocate different appointment types is surely a priority.
